# Statistical Mechanics of Zooplankton

**DOI:** 10.1371/journal.pone.0135258

**Published:** 2015-08-13

**Authors:** Peter Hinow, Ai Nihongi, J. Rudi Strickler

**Affiliations:** 1 Department of Mathematical Sciences, University of Wisconsin—Milwaukee, Milwaukee, Wisconsin, United States of America; 2 School of Freshwater Sciences, Global Water Center, University of Wisconsin—Milwaukee, Milwaukee, Wisconsin, United States of America; University of California Irvine, UNITED STATES

## Abstract

Statistical mechanics provides the link between microscopic properties of many-particle systems and macroscopic properties such as pressure and temperature. Observations of similar “microscopic” quantities exist for the motion of zooplankton, as well as many species of other social animals. Herein, we propose to take average squared velocities as the definition of the *“ecological temperature”* of a population under different conditions on nutrients, light, oxygen and others. We test the usefulness of this definition on observations of the crustacean zooplankton *Daphnia pulicaria*. In one set of experiments, *D. pulicaria* is infested with the pathogen *Vibrio cholerae*, the causative agent of cholera. We find that infested *D. pulicaria* under light exposure have a significantly greater ecological temperature, which puts them at a greater risk of detection by visual predators. In a second set of experiments, we observe *D. pulicaria* in cold and warm water, and in darkness and under light exposure. Overall, our ecological temperature is a good discriminator of the crustacean’s swimming behavior.

## Introduction

Statistical thermodynamics and ecology share at the fundamental level interest in the following question: what are the relations between “macroscopic” properties of a system, such as a volume of gas or an ecosystem and the behavior of its “microscopic” constituents, such as atoms, molecules or species and individuals. It is therefore not surprising that concepts from this branch of physics have found their way into the practice of ecology since the seminal works of Kerner [1] and Margalef [2] about half a century ago, see also [3] for a recent contribution. The challenges faced by this approach are formidable [4]. Unlike in physics, where macroscopic quantities such as pressure or temperature of a gas are easily measurable, macroscopic quantities for large scale ecosystems with a multitude of interacting agents are difficult to measure or sometimes even to define. As examples one can think of the production of organic compounds from atmospheric or aqueous carbon dioxide, nutrient cycling or the biodiversity of coral reefs and tropical rain forests. Striving to avoid these difficulties, herein we will work with more manageable observations of few individuals, small numbers of species and small observational volumes.

The vast majority of animals must move to find food, escape from predators, mate and spread into new habitats. Even sessile species such as bivalves and corals are motile in their early developmental stages. In some situations there are external sources of guidance (such as the sun, the earth’s magnetic field, acoustic or visual cues), but more often, an animal or a community of animals are searching for a specific target without knowledge of the target’s location. Millions of years of evolution have resulted in search strategies that are optimal with respect to minimal energetic cost and maximal energetic or proliferative gain. Biologists have studied the motion of countless species of the animal and protist kingdoms. There is a wealth of two-dimensional and three-dimensional positional data, both for individuals and communities of animals. Despite groundbreaking work by Okubo and others [5], it is still a challenge to analyze and interpret such data at the population level bearing in mind the important requirement of scale invariance [6]. Guided by the connections between observations on large and on small scales in physics, here we propose to apply the formalism of statistical mechanics to observations of animal motion. Our particular area of interest is zooplankton which forms a critical link between autotrophic phytoplankton and higher organisms in aquatic food webs worldwide. Earlier works studying the behavior of the crustacean *Daphnia pulicaria* [7, 8] have used power spectra of velocity autocorrelation functions and fractal dimensions [9] of trajectories to characterize swimming behavior under different environmental conditions, such as temperature, light, and possible infestation with pathogens. Although this approach allows to detect clear distinctions in motion patterns, these notions are difficult to define, to compute and often also to interpret. The goal of this work is to provide a simple method to process positional data of animal movements.

A common observational approach is to determine distance distributions for moving individuals. These are the lengths or travel times of more or less straight movements between subsequent turns. Beginning with the work [10] (in albatrosses), it has been reported in many cases that these distributions *p*(*l*) of jumps of length *l* follow power laws, that is for some constant *μ* > 0

$$\begin{array}{c} p\left(l\right)\propto l^{-\mu }, \end{array}$$ (1)

for large jump sizes, see for example [11, 12] and the references therein. The defining feature of such Lévy flights, a term coined by Mandelbrot in [13], is that the mean squared displacement 〈*x* ^2^(*t*)〉 of the walker grows faster than linear with respect to time *t*, namely

$$\begin{array}{c} \left\langle x^{2}\left(t\right)\right\rangle \propto t^{\beta }, \end{array}$$

where *β* > 1. This is in contrast to “standard” random walk, Brownian motion, where *β* = 1. (The number *H* with 2*H* = *β* is commonly known as the Hurst exponent.) The Lévy flight is more efficient than standard Brownian motion in dilute and patchy environments, since it increases the chance of leaving a largely empty region [12, 14]. On the other hand, in an environment uniformly rich in targets (for example a high concentration of algae for a feeding copepod), the walkers are observed to return to the Brownian walk [15]. It is easy to see that environmental factors such as target density and the density of the searchers critically influence the behavior of the organisms.

A major difficulty in studying searching behavior pointed out by Viswanathan *et al.* [12] is that distance distributions are difficult to determine over a sufficiently wide range such that scaling laws such as (1) can be safely established. As Viswanathan *et al.* write [12] “*For biological systems in general and foraging dynamics in particular, even 2 orders of magnitude of scaling can be a luxury.*”. In this work, we look at positional data in a way that avoids the issue of distributions on short supports. We average the squared velocities and define the *“ecological temperature”*, following an early suggestion by Margalef in [16]. We propose that this ecological temperature reacts to different environmental conditions and further to changes in environmental conditions. As an example for our paradigm, we study the behavior of the crustacean *Daphnia pulicaria* [7, 8] in cold and warm water, under conditions of light and darkness and with possible infestation by the parasite *Vibrio cholerae*. Many parasites are known to induce behavioral changes in their hosts to further their spread [17]. *V. cholerae* as the causative agent of cholera is of course a pathogen with important implications on human health. Here we review briefly important concepts from statistical mechanics and thermodynamics, focusing on the Ideal Gas Law and the equivalence of temperature and average kinetic energy. We apply these concepts to existing positional data of *D. pulicaria* [7, 8]. Finally, we discuss our results and further analogies with the thermodynamic formalism.

## Statistical Mechanics and Thermodynamics

Statistical mechanics derives macroscopic properties of a multi-particle system such as temperature and pressure from the microscopic motion of its constituents [18]. The pioneering work is connected with names such as Robert Boyle, Joseph Louis Gay-Lussac and Amadeo Avogadro. Between the seventeenth and early nineteenth centuries, these scientists established the various proportionality relations that are summarized in the empirical *Ideal Gas Law*

$$\begin{array}{c} PV=nk_{B}T. \end{array}$$ (2)

Here *P*, *V* and *T* are the pressure, volume and absolute temperature, respectively of an ideal gas of *n* molecules. The constant *k*_*B*_ is called Boltzmann’s constant. In the 1860s, James Clerk Maxwell and Ludwig Boltzmann finalized the theory of thermodynamics. In the simplest model of a monatomic gas, *n* identical molecules of mass *m* are moving in a fixed volume *V* subject only to elastic binary collisions. Under the assumption that velocity components in the three directions are independent, it can be derived that the average kinetic energy of the gas particles is proportional to the temperature,

$$\begin{array}{c} \frac{1}{2}m\left\langle v^{2}\right\rangle =\frac{3}{2}k_{B}T. \end{array}$$ (3)

A great achievement of statistical mechanics is precisely the connection between the macroscopic empirical law (2) and the microscopic average kinetic energy in (3). Thus, if there were no thermometers to measure the temperature of a gas, Eq (3) would give an alternative method: ***measure the average squared velocity of the particles***. The question now is, can we use this analogy for other agents?

It bears repeating that the Eq (3) was derived under certain well-defined conditions. These are that the particles follow the laws of classical Newtonian physics, so neither quantum nor relativity theory are needed. The gas has to be in equilibrium near the standard conditions of a temperature of 273 *K* and a pressure of 100 *kPa*. This implies large numbers of particles, of the order of Avogadro’s constant *N*_*A*_ = 6.02 ⋅ 10^23^ *mol* ^−1^. However, these conditions are not restrictive and from this derives the importance of the “Naturkonstante” *k*_*B*_ (so called by Max Planck). A more detailed treatment would include that kinetic energy can also be contained in vibrations and rotations of chemical bonds between atoms. In a non-equilibrium state of the gas there can be heating or cooling or a motion of the center of mass. This leads to the formulations of First and Second Laws of Thermodynamics.

We propose to determine experimentally the average squared velocity for autotrophic and heterotrophic plankton communities. Led by the analogy with Eq (3) we call this the “ecological temperature”. To begin with, we consider populations that have constant conditions of nutrient supply, light and water temperature and can therefore be regarded to be in an equilibrium state.

## Materials and Methods

### Experimental Design

*Daphnia pulicaria* were observed with and without infestation with *Vibrio cholerae* in an experimental aquarium of 80 *mL* at water temperature of 22°*C*. For a detailed description of the experiments we refer to [8]. Under every combination of the conditions, ten animals were observed, except in the case of infested individuals under light, where only nine animals were observed for 15 *min* at a frame frequency of 30 *Hz*. This results in 39 data sequences, each of length *N* ≈ 2.7 ⋅ 10^4^. In a second set of experiments [7], *D. pulicaria* are placed in lake water at 3°*C* and at 22°*C*, while the light condition is also varied. Five observations are made in each of the four cases. The original data are available as supporting information to this paper.

### Data Analysis

We assume that a population consists of *n* identical individuals and that (initially at least) the conditions are spatially uniform and do not change during the course of the observation. The data are given in form of sequences ${\left(X_{i}^{k}\right)}_{i=1}^{N_{k}}$ where *k* = 1, …, *n* denotes the index of the sequence (the animal), and *X* denotes a point (*x*, *y*) or (*x*, *y*, *z*). Sequences can be of different lengths. For sake of simplicity we will consider the case that the positional data are two-dimensional, with the obvious modifications for three-dimensional data. The sampling rate is *f* and *τ* = *f* ^−1^ is the time between two frames. We compute the squared velocities

$$\begin{array}{c} {\left(V_{i}^{k}\right)}^{2}\;=\frac{{\left(x_{i+1}^{k}-x_{i}^{k}\right)}^{2}+{\left(y_{i+1}^{k}-y_{i}^{k}\right)}^{2}}{\tau^{2}},\;i=1,\ldots ,N_{k},\;k=1,\ldots n. \end{array}$$

Then we compute the *orbit averages* along a trajectory of a fixed animal

$$\begin{array}{c} {\left({\bar{V}}^{k}\right)}^{2}=\frac{1}{N_{k}}\sum_{i=1}^{N_{k}}{\left(V_{i}^{k}\right)}^{2},\;k=1,\ldots ,n. \end{array}$$ (4)

We obtain an empirical distribution of values for the different individuals, under the same experimental condition. Here we make the hypothesis of *ergodicity* of the population: *Every individual experiences the environment in the same way and so the orbit averages are manifestations of a variable that characterizes the population.* We average again and define the ecological temperature

$$\begin{array}{c} {\left(\bar{V}\right)}^{2}=\frac{1}{n}\sum_{k=1}^{n}{\left({\bar{V}}^{k}\right)}^{2} \end{array}$$ (5)

of the population under a certain environmental condition.

To test for differences between populations under different experimental conditions, we use the Kolmogorov-Smirnov test [19]. For a given set of values *x*_1_, …, *x*_*m*_ (these are not to be confused with the positions of animals used earlier), we define the empirical cumulative distribution function

$$\begin{array}{c} F\left(x\right)=\frac{1}{m}|\left\{x_{i}\;:\;x_{i}\leq x\right\}|. \end{array}$$ (6)

Denoting by *F*_1_ and *F*_2_ the cumulative distribution functions under the different experimental conditions 1 and 2, respectively, the value of the Kolmogorov-Smirnov statistic is

$$\begin{array}{c} D=\underset{x}{\sup}\left|F_{1}\left(x\right)-F_{2}\left(x\right)\right|. \end{array}$$

Let

$$\begin{array}{c} Q\left(\lambda \right)=2\sum_{j=1}^{\infty }{\left(-1\right)}^{j-1}\exp\left(-2j^{2}\lambda^{2}\right), \end{array}$$

this is a monotone decreasing function with

$$\begin{array}{c} Q\left(0\right)=1,\;\text{and}\;\lim_{\lambda \to \infty }Q\left(\lambda \right)=0. \end{array}$$

The significance of an observed value of *D* as a disproof of the null hypothesis

$$\begin{array}{c} H_{0}:\;\text{The two distributions}\;F_{1}\;\text{and}\;F_{2}\;\text{are equal}. \end{array}$$

is given by

$$\begin{array}{c} P\left(D>\text{observed}\right)=Q(\sqrt{\frac{m_{1}m_{2}}{m_{1}+m_{2}}}), \end{array}$$

where *m*_1_ and *m*_2_ are the number of data points in each distribution (the number of animals observed under each condition). The significance of this test arises from large numbers of observations *m*_1_ and *m*_2_.

## Results

*Daphnia* are crustaceans that feed on suspended materials (e.g. algae and organic detritus) in freshwater environments [20]. We use existing two-dimensional observations of *D. pulicaria* as presented in Nihongi *et al.* [8]. Here the differences in environmental conditions are light (light vs. dark) and a possible infestation with *Vibrio cholerae*, giving a 2 × 2-matrix of conditions.

**Fig 1:**
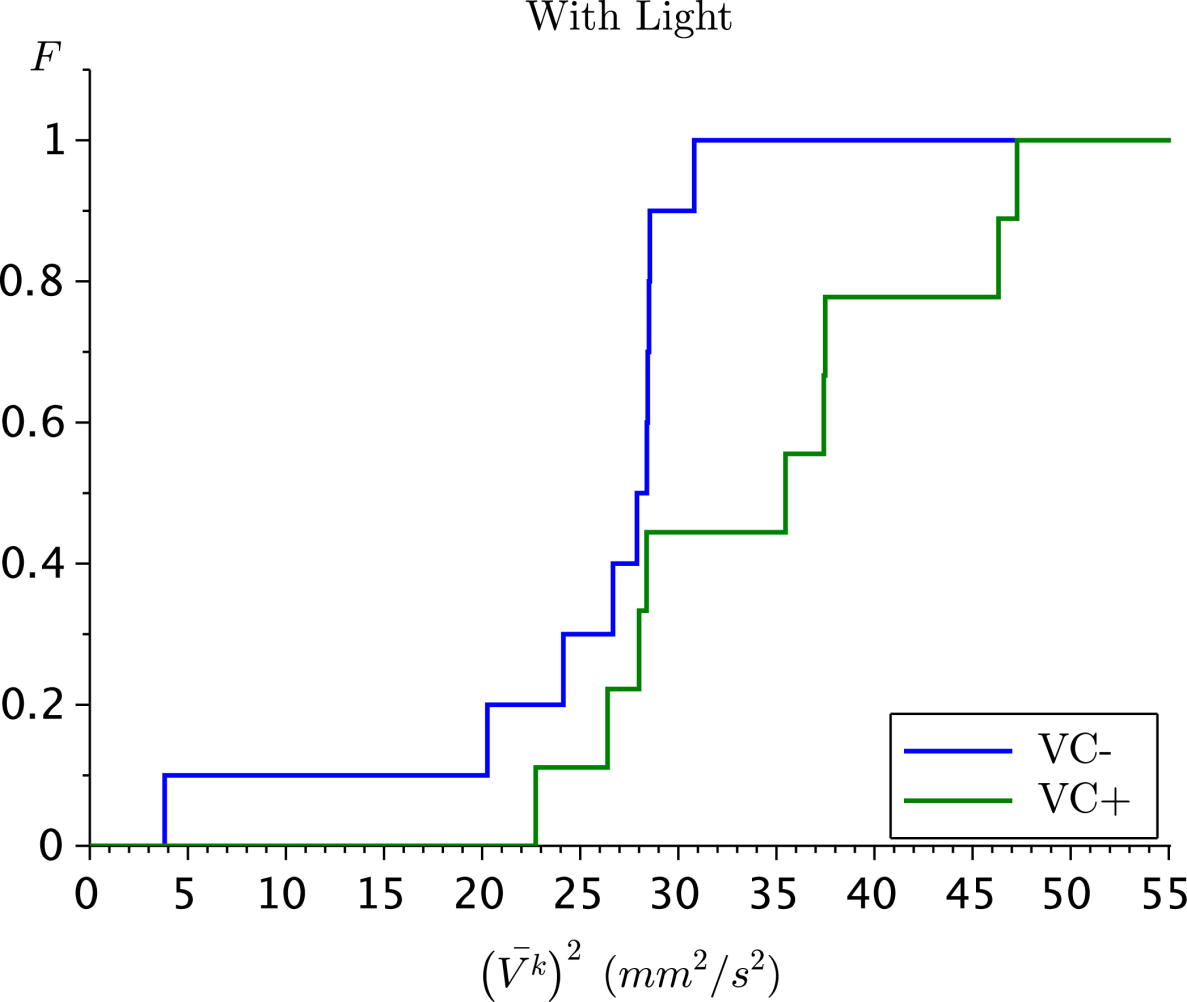
The cumulative distribution functions (see Eq (6)) of the orbit averages (see Eq (4)) of ten infested (red) respectively nine uninfested (blue) *D. pulicaria* in presence of light. For ease of reading, the other cumulative distribution functions are not shown.

In Fig 1 we show the cumulative distribution functions of the orbit averages ${\left({\bar{V}}^{k}\right)}^{2}$ in the presence of light for both infested and uninfested *D. pulicaria*. The hypothesis of equality of these distributions can be rejected at a confidence level of *α* = 0.1. In the other cases there was no statistically significant difference in the cumulative distributions. In Table 1 we report the values of ${\left(\bar{V}\right)}^{2}$ together with their standard deviations for all four scenarios. We observe the largest value of ${\left(\bar{V}\right)}^{2}$ for infested *D. pulicaria* under light exposure. This is consistent with the findings of Nihongi *et al.* [8] where similar findings were made based on the power spectra of the swimming velocity fluctuations.

**Table 1:** Ecological temperature and its standard deviation under different combinations of the conditions.

Light	Infestation Status	${\left(\bar{V}\right)}^{2}$ (in *mm* ^2^/*s* ^2^)	*σ*
-	-	28.9	16.3
-	+	27.5	12.8
+	-	25.2	13.8
+	+	33.1	14.8

**Fig 2:**
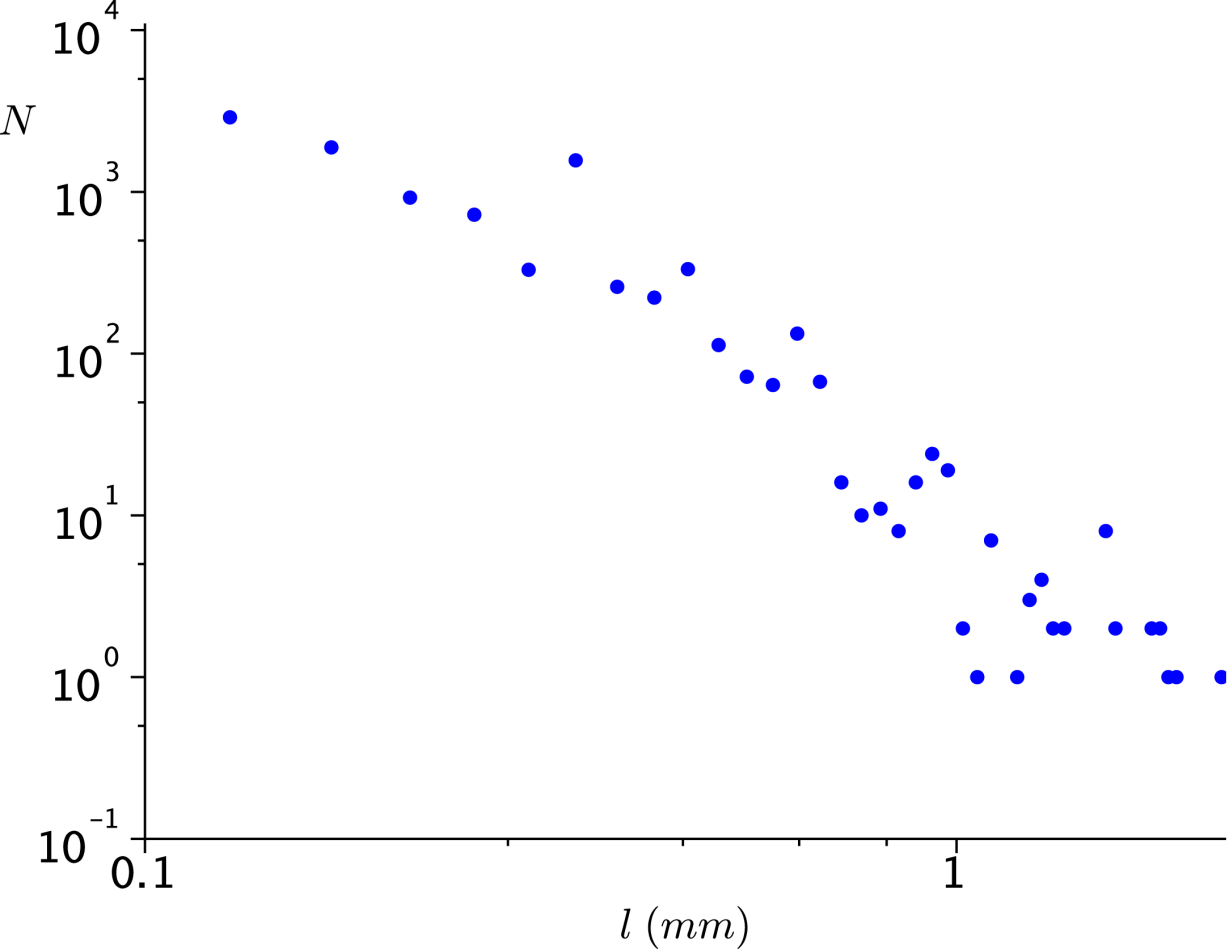
A sample jump length distribution for an infested individual under light exposure. However, 42% of the jumps had length zero. The non-zero jumps range from only 100 *μm* to 4.2 *mm*, too short to fit any distribution (exponential or power law) to it.

In Fig 2 we show an example of a distribution of nonzero jumps for one infested *Daphnia* individual under light. This animal was selected for making the single longest jump of 4.2 *mm* (approximately four body lengths). In fact, 42% of the jumps of the individual had length zero. It is difficult to decide about the existence of a heavy tail in this distribution since it covers a short range of lengths.

The Gram-negative bacterium *Vibrio cholerae* is the cause of the endemic disease cholera which affects an estimated 3–5 million people and causes 100,000–130,000 deaths annually [21]. It is known to adhere to the chitin exoskeleton of zooplankton [22, 23]. Using our concept of ecological temperature, we have provided further evidence that *V. cholerae* increases the activity of its host *D. pulicaria* under light conditions. This contributes to the theory that such individuals are more likely to be detected and captured by visual predators such as fish. This can be due to two reasons that are non-exclusive. First, the parasite enters the aquatic food chain and so improves its dispersal. Second, the infested *D. pulicaria* are eliminated from the population and so reduce the chance of other individuals becoming infested. In some sense, this is a win for both the parasite and the host.

**Fig 3:**
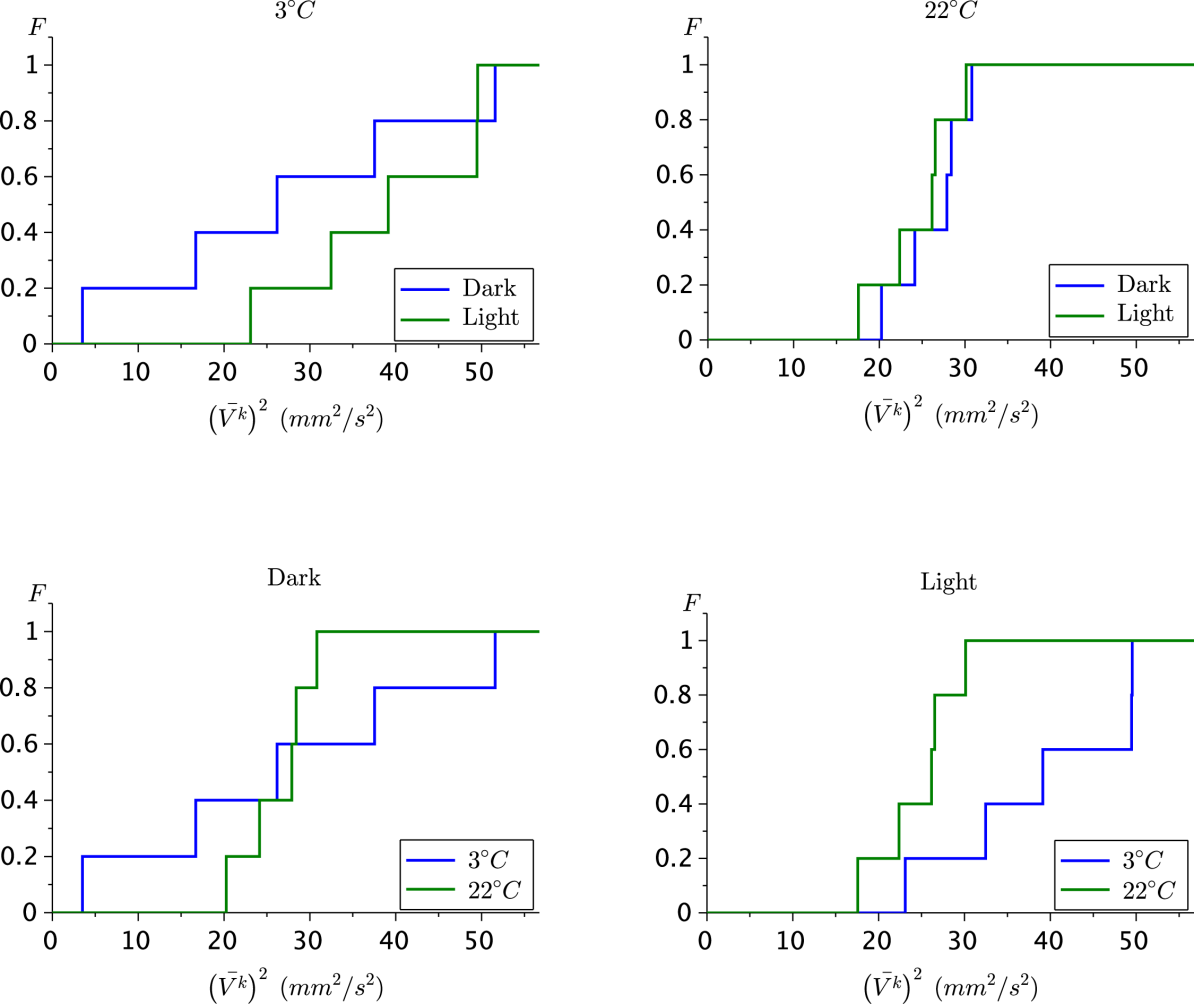
Cumulative distribution functions of the ecological temperature. The individuals are placed in cold and warm water and exposed to light or darkness. In each panel we compare the cumulative distribution functions when the value of one variable changes.

In the second set of experiments from [7], we compute the ecological temperature for *D. pulicaria* individuals in cold (3°*C*) and warm (22°*C*) water in darkness and light. Since there are only five observations in each case, we refrain from statistical hypothesis testing. However, we can state the following, see Fig 3.

1. At 3°*C*, turning the light on increases the ecological temperature significantly, while at 22°*C*, there is practically no difference between light and dark conditions.
2. In darkness, the individuals tend to behave much more differently from one another in cold water than in warm water.
3. Under light, there is a significantly higher ecological temperature in cold water than in warm water.

These differences in behavior can be interpreted as the result of adaptation to phytoplankton availability and distribution and to predator behavior in lakes in winter and summer and during night and day.

In future experiments we will vary the communal and environmental conditions. These are for example

- rich or poor conditions of supplies such as nutrient, light and oxygen,
- high or low population density,
- environmental conditions such as ambient temperature, presssure and pH,
- presence of chemicals, in particular toxins,
- presence of predators, pathogens or competing species, and
- mating searches [24].

Our objects of study include protists (ciliates, dinoflagellates), small multicellular zooplankton (rotifers) and planktonic crustaceans (*Daphnia*, copepods). We will seek for differences between repeated individual and community observations. Animals may make active moves to avoid each other. Is there evidence for cooperation among the searchers intermediate densities, resulting in higher ecological temperature?

Many planktonic organisms are known to display phototaxis [25, 26] and chemotaxis [27, 28]. Organisms such as *Paramecium* and *Daphnia* are also used as biological indicators to test for toxins and nano-particles [29] in water bodies intended for human use. Current observation methods either rely on complicated properties of trajectories (velocity power spectra, fractal dimensions of paths, [7, 8]) or physiological observations (heart or appendage beating frequencies, [29]) that are often difficult to interpret or reproduce. The ecological temperature as defined in this paper has the advantage of great conceptual and computational simplicity and draws on the analogy with well-known concepts from statistical mechanics.

## Outlook

Once a clear difference in the activity from Eq (5) has been established for different conditions, it will be very interesting to simulate phase transitions between these conditions. For example, if the light is turned on, how long does it take for the plankton community to adapt to it? Even switching back and forth is feasible. This gives the possibility to study learning in some of the 95% of animal species that are invertebrates [30].

A later and more challenging setup will allow for “binary” environments. Assume that the regions *A* and *B* are distinguished by the value of the environmental variable in them (such as adjacent regions of light and darkness). It is possible that a trajectory often switches between these two regions. For such a trajectory the orbit average would not be very meaningful and therefore it should be discarded. Most interesting are the trajectories that pass the boundary between *A* and *B* exactly once (in either direction). For such trajectories we can determine the orbit average for each of the two segments in the different regions. How often do we see a marked difference between these?

**Fig 4:**
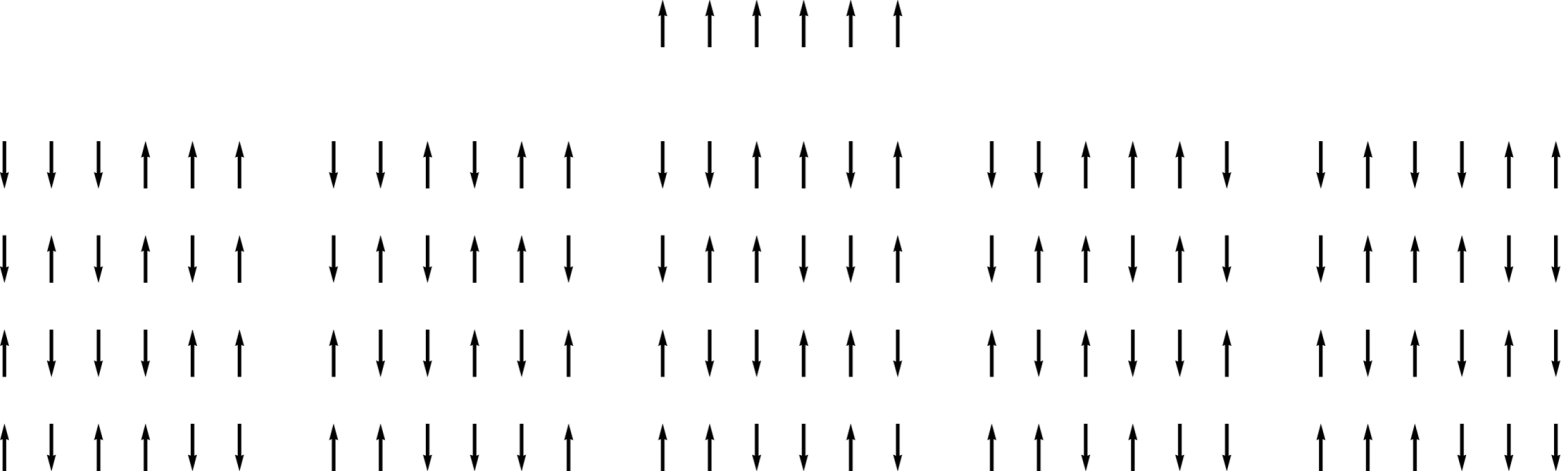
Macrostates of low *(top)* and high *(bottom)* disorder in two sets of directed particles or molecular axes. Disorder is clearly realized by many more microstates. If subject to random, thermal motion, the system will evolve only from the state of high order to the state of low order. To create order, a source of energy is required.

Concepts from statistical mechanics have been used quite frequently to understand the collective behavior of animals such as schools of fish and flocks of birds [3, 31]. In future work we will look beyond the notion of ecological temperature as it has been introduced here. The *entropy* *S* of a physical system is proportional to the logarithm of the number of microscopic states that realize one and the same macroscopic state (The equation *S* = *k* log *W* is engraved on Boltzmann’s tombstone at the Zentralfriedhof in Vienna.). For example, consider the Ising model of ferromagnetism with a small number of states. Assume that 6 arrows can point up (+1) or down (-1) (these are the “microstates”), but that we are only interested their sum (this is the “macrostate”). The macrostate 6 can be realized by only one microstate (1, 1, 1, 1, 1, 1), whereas the macrostate 0 can be realized in $\left(\begin{array}{c} 6\\ 3 \end{array}\right)=20$ ways, among them for example (−1, 1, 1, 1, −1, −1) or (1, −1, 1, −1, −1, 1), see Fig 4. Thus we assign the entropy 0 respectively log20 to both these states. The presence of the logarithm is necessary to make the entropy an extensive property, that is, if two subsystems are united, the entropy of the resulting system is the sum of the entropies of the subsystems. The entropy can be interpreted as a measure for the lack of order in a thermodynamical system. The Second Law of Thermodynamics states that in a closed system the entropy necessarily increases. Clearly, many systems in nature are not closed, since they receive energy from external sources.

In the case of the animals, we search for an analogous measure for order respectively disorder in motion patterns. A well-known example is the diurnal vertical migration of zooplankton communities. Organisms move upwards at night and downwards during the day. The causes of this migration are manifold, such as finding regions of optimal light intensity, optimal temperature and predator avoidance [32]. The general movement patterns may be disturbed by the presence of visual or tactile predators. The conventional view has been that each individual migrates on its own, without need for cues from conspecifics. The challenge arises whether the statistical mechanics approach is capable to detect signs of independence or coherence in the animal behaviors.

## Supporting Information

S1 Data Files Raw data of *Daphnia pulicaria* under possible infestation with *Vibrio cholerae* under conditions of light and darkness. Information on the file contents and the data format is contained.
(ZIP)

S2 Data Files Raw data of *Daphnia pulicaria* in warm and cold water and in light and dark conditions. (ZIP)
